# The Role of Empathic Concern and Gender on Interspecific Contagious Yawning in Humans

**DOI:** 10.3390/ani13101700

**Published:** 2023-05-20

**Authors:** Andrew C. Gallup, Sabina M. Wozny

**Affiliations:** Psychology and Evolutionary Behavioral Sciences Programs, SUNY Polytechnic Institute, Utica, NY 13502, USA

**Keywords:** biobehavioral synchrony, emotional contagion, empathy, human–animal bond, human–animal interaction, sex differences

## Abstract

**Simple Summary:**

Contagious yawning (CY) has garnered considerable interest in the fields of psychology and animal behavior, particularly as it relates to the potential connection this response has with empathy or emotional contagion. Recent reports have explored this association through the study of interspecific CY, whereby the detection of yawns from one species trigger yawning in a different species. While the evidence for interspecific CY is robust, links to empathy have been inconsistent. Here, we sought to explore this relationship more explicitly in humans by assessing how interspecific CY in response to images of yawns from common household pets relates to individual differences in empathic concern. The results provide further evidence for interspecific CY in humans, but self-reported empathic concern was a negative predictor of this response. We also found no sex difference in interspecific CY, though when comparing the sexes across the CY conditions, women reported a higher frequency of yawning in response to dog yawns, and men reported a higher frequency of yawning in response to cat yawns. Overall, these findings do not support a strong connection between interspecific CY and empathy or emotional contagion.

**Abstract:**

Interspecific contagious yawning (CY), whereby yawns from one species trigger yawning in different species, has now been reported across various taxa. This response to human yawning appears common among animals in captivity and has been interpreted as an empathic response towards human handlers/caregivers. A recent study found that humans also display interspecific CY, though this response was not modulated by proxies of empathic processing (i.e., phylogenetic relatedness or social closeness to the animals). Here, we explored this relationship more explicitly by assessing how interspecific CY to yawns from common household pets relates to self-reported empathic concern. Participants (*N* = 103) completed a survey measuring empathic concern and then reported on their yawning behavior following exposure to a control condition or yawning images either from domesticated cats or domesticated dogs. The results provide further evidence for interspecific CY in humans, but empathic concern was negatively predictive of this response. There was also no sex difference in interspecific CY, though when comparing the sexes across CY conditions, women reported a higher frequency of yawning in response to dog yawns, and men reported a higher frequency of yawning in response to cat yawns. Overall, these findings do not support a strong connection between interspecific CY and empathy or emotional contagion.

## 1. Introduction

Contagious yawning (CY) is a distinctive pattern of behavioral coupling that occurs when the mere detection of yawns by others elicits the automatic and reflexive tendency for an observer to yawn. Yawning can be reliably elicited in this way in humans using images, videos, or audio clips of yawning [1,2,3,4,5], though there is considerable variability in this response [6,7]. While the first experimental demonstration of CY in a nonhuman animal was published less than two decades ago [8], evidence for CY is now present for a growing number of social vertebrates (for a review, see [9]). Various theories have been proposed pertaining to the biological significance of CY [10], with recent evidence indicating a role in facilitating motor synchrony and enhancing vigilance in groups [11,12].

Independent of its ultimate function, one view that has gained considerable traction in the scientific community is that CY represents a primitive form of empathic processing, i.e., the tendency to yawn after detecting this action in another could reflect a form of emotional contagion. This idea, which could explain the distinctive individual differences in CY across studies, was initially proposed in a monograph by Lehmann [13] and further elaborated within the perception–action model of empathy [14,15]. Empirical support for a connection between empathy and CY was first published by Platek et al. [6], and this continues to be a prevailing view in the current literature [16]. However, systematic reviews of the studies examining the linkage between CY and empathy have shown that the totality of evidence is rather mixed and inconsistent [17]. Alternatively, CY could instead represent a simple feature of behavioral contagion or facial mimicry [18,19] that would show a statistical correlation to markers of empathy or emotional contagion without a causal relation. For example, one key issue in this debate is that at a proximate level CY requires the detection of yawns in others, and social attention and empathy are often difficult to disentangle [20,21]. Thus, while the use of CY as a potential indicator of empathy or emotional contagion is intriguing, a direct linkage remains unclear.

Recently, interspecific CY, whereby yawns from one species trigger a member of a different species to yawn, has been examined in relation to empathy. The first evidence for interspecific CY came 15 years ago from a study showing that the overt display of human yawning was sufficient to induce a similar response among domesticated dogs [22]. This initial report garnered considerable attention and led to numerous follow-up studies designed to explore the interspecific CY/empathy connection. If this cross-species facial mimicry was enhanced by empathy, it had been predicted that dogs should respond more strongly to yawns from caregivers/owners where there was an existing social bond or attachment. This view was based largely on a 2011 study of chimpanzees, whereby an ingroup bias for CY was observed and interpreted as a sign of empathy [23]. The authors of this work suggested that since humans show ingroup biases for empathy for pain [24,25,26], similar biases in CY could be used as a measure of affective empathy. Overall, the evidence in support of this effect among dogs is mixed [27,28,29], and a recent study, including a meta-analysis of this literature, concluded that human-initiated CY in dogs was, in fact, not a signal of empathy [30].

Studies on other captive species, however, have reported more consistent familiarity/ingroup biases when it comes to interspecific CY. In line with the view that CY may reflect a form of empathy or emotional contagion, familiarity has been shown to enhance both intra- and interspecific CY among nonhuman primates [23,31,32]. In one study on chimpanzees, subjects responded with CY to stimuli from familiar humans (i.e., researchers and husbandry staff) and ingroup conspecifics but not to outgroup chimps or unfamiliar members from other species [31]. A more recent and very similar study on captive red-capped mangabeys produced the same basic findings: subjects showed a higher CY response to familiar human caretakers and conspecifics compared to unfamiliar individuals from across three species [32]. In addition, one study on African elephants found that individuals responded with CY to yawns from familiar human handlers [33], though no comparisons were made with unfamiliar humans.

To further examine this relationship, Gallup and Wozny [5] investigated whether humans also display interspecific CY. Using an online format, participants were shown a series of yawning stimuli from one of the following categories: fish, amphibians, reptiles, birds, non-primate mammals, great apes, and a compilation of common household pets (including an equal representation of domesticated cats and dogs). Given that prior studies have shown that people display greater empathy and attachment towards both more closely related species [34,35,36] and domesticated animals that are commonly kept as pets [37,38,39], it was predicted that interspecific CY would be enhanced by phylogenetic proximity and domestication/social closeness. The findings provided clear evidence for interspecific CY when compared to the control images, with 69% of participants reporting interspecific CY. Yet, while it was expected that yawns from great apes (chimpanzees, gorillas, and orangutans) and common pets (cats and dogs) would elicit the greatest response, this was not the case. Consequently, these findings did not support the purported connection between interspecific CY and empathy or emotional contagion in humans and instead suggest that the mechanisms governing CY are generalized. Importantly, Gallup and Wozny [5] included a task that ensured attention towards the yawning stimuli during testing. Given CY is a reflexive and automatic response, it seems that the detection of yawns, independent of the taxa represented, may be sufficient to generate this response. However, like previous works, overt measures of empathy or attitudes towards animals were not obtained in this experiment. In addition, subsequent exploratory analyses from this open-access dataset indicated that women may be more likely to yawn in response to yawns from a compilation of CY stimuli from domesticated cats and dogs. While sex differences in CY are rarely observed (for a review, see [40]), and an overall effect of gender was not found. In general, it has been argued that a female bias in CY would be consistent with links to empathy and/or emotional contagion [41]. Thus, future research could help clarify this picture by assessing whether this interaction is reproducible.

The current study served as a direct follow-up to Gallup and Wozny [5], providing a more explicit examination of the relationship between interspecific CY and empathy in humans. We sought to examine whether validated measures of empathic concern and animal attitudes predicted interspecific CY to common household pets while controlling for attention. Empathic concern was selected because it has previously been shown to correlate with animal attitudes [42,43] and intraspecific CY in humans [44], while measures of cognitive empathy (i.e., reading emotions and perspective-taking) tend to be less correlated with animal attitudes and have more often failed to predict CY in humans [45,46,47]. Given that dogs have undergone a long period of domestication that involved distinct forms of social communication and cooperation with humans [48] and that people tend to perceive both human and dog facial expressions in a similar manner [49], we examined interspecific CY separately to domesticated cats and dogs. Moreover, based on exploratory analyses showing a potential female bias in interspecific CY to common household pets, we aimed to replicate this effect of participant gender. If empathy was related to interspecific CY, empathic concern should positively predict this behavior, and perhaps women would show a greater response [41]. If interspecific CY is generalized, due to it being more of an automatic and reflexive response, merely attending to the animal stimuli should elicit yawns independent of empathic concern or gender.

## 2. Materials and Methods

### 2.1. Participants

Following the methods of Gallup and Wozny [5], participant recruitment occurred online using Amazon Mechanical Turk (MTurk): https://www.mturk.com/ (accessed on 22 November 2022). This study was approved by the Institutional Review Board at SUNY Polytechnic Institute (IRB-2022-5), and informed consent was required prior to initiating the study. Several restrictions were applied to improve data quality. First, eligible MTurk workers were required to live in the United States (US) and have completed a minimum of 100 tasks, with a successful completion rate >95%. To address potential issues of reduced attention among MTurk respondents, we recruited a total of 180 participants (60 per condition) and included various attention checks [50,51]. To screen for inattentive respondents and bots [52], for example, an initial attention check question and a Completely Automated Public Turing test to tell computers and humans apart (CAPTCHA) were included within the initial demographic questions. Similar attention checks were also embedded within the empathic concern and animal attitudes survey measures (see below). Incorrect responses to these items excluded 24 participants. Moreover, given that visual detection of yawning is critical when attempting to elicit CY with videos or imagery [20,53], a dozen additional attention check questions were embedded as part of the task when reviewing the CY stimuli (see design and procedure below). Another 35 participants were excluded due to incorrect responses to these questions. An additional 17 participants were excluded because they did not complete the study, and one last participant was not included in the analysis because they chose not to identify their gender. This left a final vetted sample of 103, including 53 men and 50 women (age M ± SD: 34.7 ± 10.2).

### 2.2. Design

This study employed a between-subjects design. Like Gallup and Wozny [5], data collection occurred via Google Forms, and 24 pictures were used for each of the three conditions from online image searches (e.g., Google): domesticated cats, domesticated dogs, and control. For the two interspecific CY conditions (domesticated cats; domesticated dogs), twelve images were displayed of animals in mid-yawn (i.e., extended gaping of the jaw, head tilting, eye closure), while the other twelve images were of animals of the same or closely related breed/coloring clearly not yawning. Half of these images were previously used in Gallup and Wozny [5], including a compilation of six domesticated dog and cat images, each in one pet condition, while the other half were new. The same 24 images from the previous study were also used for the control stimulus, half consisting of open building windows and the other half depicting similar windows and frames that were shut. Maintaining the original aspect ratio, all images were then standardized to 7.62 cm in height. As in Gallup and Wozny [5], similar images of yawning and non-yawning animals or open and closed windows were paired side-by-side in randomized right/left positioning (note: all stimulus images are available upon request).

### 2.3. Procedure

Following some initial demographic questions, participants completed the seven-item empathic concern scale of the Interpersonal Reactivity Index (IRI) [54] and the five-item version of the Animal Attitude Scale (AAS-5) [55]. Internal consistency was acceptable for the empathic concern measure (α = 0.72) but was too low for the AAS-5 to be included in subsequent analyses (α = 0.27).

Next, participants were given the following instructions when viewing the CY stimuli for the condition they were assigned (dog or cat): “You are going to be presented with twelve pairs of images, one at a time, each depicting one animal that is yawning and one animal that is not. You need to review each image pairing and correctly identify the animal that is yawning by indicating whether it is the image on the left or the right. It is important to answer each question accurately” (p. 4, [5]). As we were not able to monitor or record the visual attention of participants during testing, this procedure ensured that each yawn was detected during trials. As we closely matched the animals for the yawning and non-yawning stimuli in each pairing, the participants could not rely on other stimulus characteristics to make their determination and thus were forced to review both images and correctly label the one with an animal yawning. For the control condition, instructional references were made to open and closed windows rather than yawning and non-yawning animals. Evaluation of the window images in the control condition served as a comparable task visually, but one that would not be expected to induce yawning. 

After viewing and responding to the entire stimulus compilation for the condition they were assigned, participants then self-reported on their yawning behavior (yes/no and how many times), which has been shown to be a valid measure of CY [3,56,57]. Lastly, based on the association between yawning and sleep and fatigue [58,59], participants indicated their sleep duration (in hours) the previous night and how tired they felt on a 10-point scale (1: not tired at all; 10: extremely tired).

### 2.4. Analysis

The final sample included 39 participants in the cat yawning condition, 27 participants in the dog yawning condition, and 37 participants in the control condition. A post hoc power analysis was performed using G*Power 3.1 [60] with a medium effect, revealing power of 0.784 and 0.811 to detect a main effect for the binary and frequency measures of yawning, respectively. A binary logistic generalized linear model (GLM) was run for yawn occurrence, while a Poisson loglinear GLM was run for yawn frequency. Each model included stimulus condition and participant gender as factors, and the output included stimulus × gender interactions. Participant age, prior sleep, current tiredness, and the self-reported trait measure of empathic concern were included as covariates. All analyses were conducted in jamovi [61]. 

## 3. Results

In total, 57.6% of participants reported yawning in response to the interspecific CY stimuli, with an average of 2.88 ± 3.86 yawns/participant. In the control condition, 54.1% of participants reported yawning, with an average of 2.41 ± 3.16 yawns/participant. Table 1 includes the descriptive statistics for the non-yawning variables.

**Figure 1:**
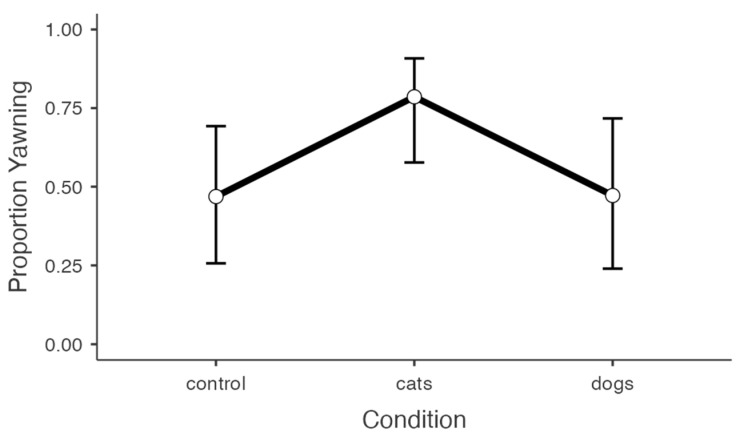
Line graph plot depicting the proportion of yawners across conditions. Data are presented as estimated marginal *M* ± 95% CI.

**Figure 2:**
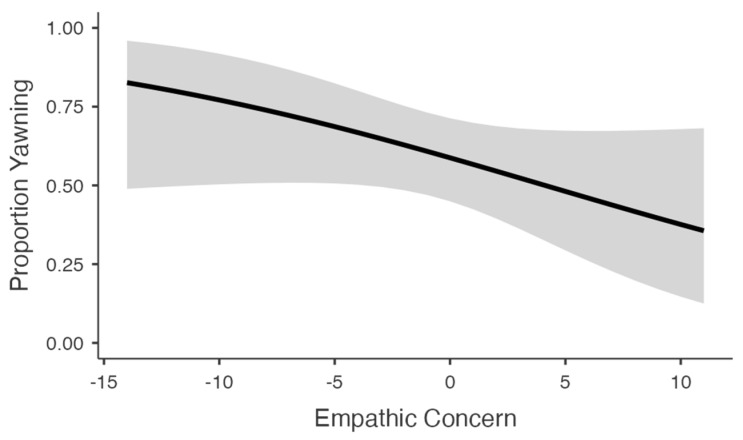
Simple slopes plot depicting the relationship between empathic concern and the tendency to yawn during testing. The shaded region represents 95% CI.

For yawn occurrence (yes/no), prior sleep duration and current tiredness were both significant predictors (Table 2). Overall, participants who reported sleeping for fewer hours the previous night and being more tired at the time of testing were more likely to report yawning while reviewing the stimuli (*ps* < 0.01). There was only a marginal effect of stimulus condition (*p* = 0.059), with planned comparisons showing that participants in the cat yawning condition were significantly more likely to yawn compared to the control condition (*p* = 0.046; Figure 1). No other comparisons were significant (*ps* > 0.05). Empathic concern was not a significant predictor (*p* = 0.122), though the trend was in the opposite direction from what would be expected, i.e., individuals that scored higher on self-reported empathic concern were less likely to yawn (Figure 2). When assessing just the CY conditions, we found that participants that yawned scored significantly lower in empathic concern (Welch’s *t_66_* = 2.23, *p* = 0.031). No effect was observed for the control condition (Welch’s *t_37_* = 1.64, *p* = 0.118). Lastly, there was no main effect of participant gender (*p* = 0.851), nor was there a condition × gender interaction (*p* = 0.538). 

**Figure 3:**
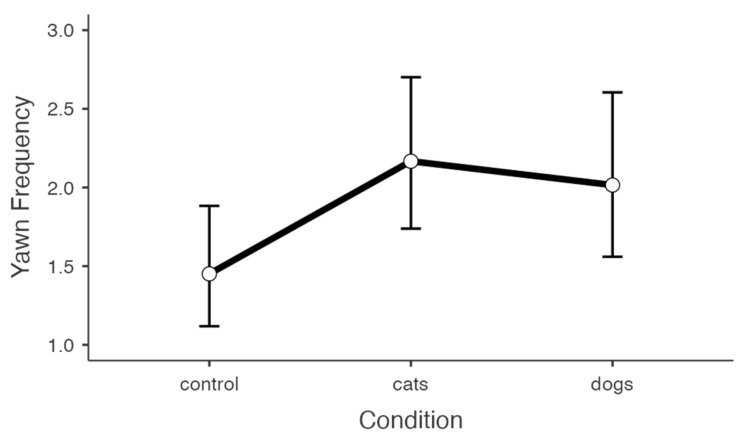
Line graph plot depicting yawn frequency across conditions. Data are presented as estimated marginal *M* ± 95% CI.

**Figure 4:**
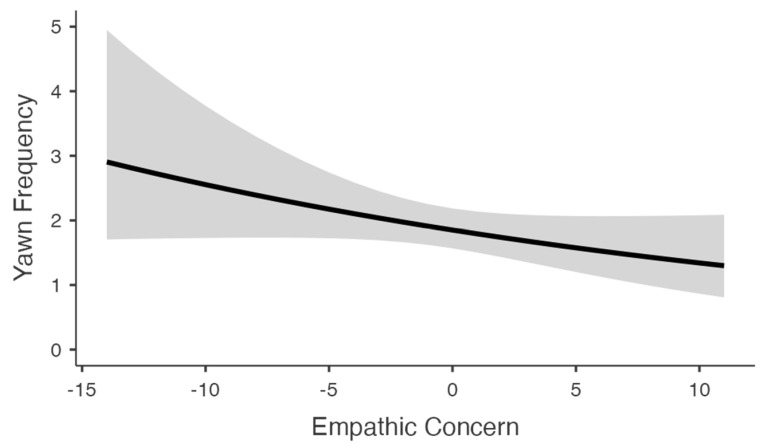
Simple slopes plot depicting the relationship between empathic concern and yawn frequency during testing. The shaded region represents 95% CI.

**Figure 5:**
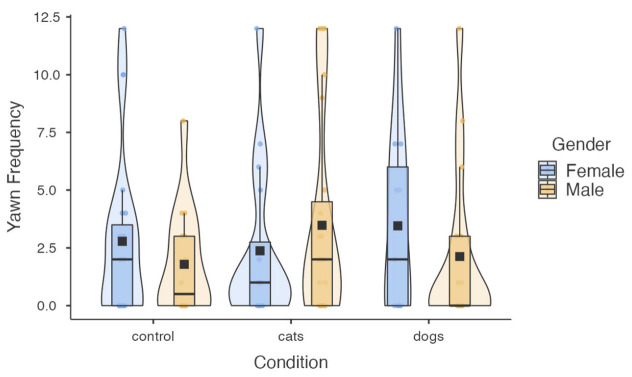
Yawn frequency as a function of stimulus condition and gender. Box plots represent the median, interquartile ranges, and the whiskers extend 1.5 times the interquartile range for the upper and lower boundary. Violin plots illustrate the distribution of yawning. Observed responses are depicted by blue and orange circles, and group means are depicted by black boxes.

For yawn frequency, stimulus condition, prior hours of sleep, and current tiredness were all significant predictors (Table 3). Like the binomial model, participants who indicated sleeping for shorter durations the previous night and feeling more tired at the time of testing reported more yawns (*ps* < 0.01). In this case, however, participants in both yawning conditions reported more yawns compared to the control condition (main effect: *p* = 0.023; Figure 3), though this effect was marginal for the dog condition (cat: *p* = 0.009; dog: *p* = 0.054). No difference in the frequency of interspecific CY emerged between the cat and dog conditions (*p* = 0.645). Like the binomial model, there was a trend for empathic concern whereby individuals that scored higher on this measure reported fewer yawns (*p* = 0.098; Figure 4). When assessing just the CY conditions, we found a significant negative correlation between empathic concern and self-reported yawn frequency (*B*_66_ = −0.278, *p* = 0.003). No correlation was observed for the control condition (*B*_37_ = −0.193, *p* = 0.135). Lastly, there was no main effect of participant gender, but a significant stimulus condition × gender interaction emerged (*p* = 0.005). When comparing the sexes, women reported greater interspecific CY to images of dog yawns, and men reported greater interspecific CY to images of cat yawns (Figure 5). 

## 4. Discussion

The current findings provide further evidence of interspecific CY in humans, replicating previous research [5]. While the magnitude of this effect was relatively limited for yawning images of domesticated dogs, showing up only when examining CY frequency, there was a larger and significant effect observed within the cat yawning condition across both the binary and frequency models. This difference in interspecific CY when viewing images of cat and dog yawns was not expected given the coevolution of human–dog bonding and social communication [48,49,61]. Nonetheless, these results, coupled with the report of Gallup and Wozny [5], provide strong support that, in general, yawns from common household pets can reliably elicit CY among humans. 

A primary objective of the current study, outside of replicating interspecific CY in humans, was to more explicitly assess how this response varied as a function of individual differences in a self-reported measure of affective empathy. To date, prior studies in this area have only used proxies for empathy, focusing on features pertaining to the familiarity (e.g., human handlers/caregivers) or phylogenetic closeness of the interspecific stimuli. Here, we had participants complete the empathic concern subscale of the IRI [54] as well as the five-item version of the Animal Attitude Scale (AAS-5) [55] to assess how this predicted interspecific CY. Although the reliability of the AAS-5 was poor, the internal consistency of the empathic concern scale was acceptable and included in the analysis. Moreover, this measure of affective empathy towards humans has previously been shown to predict attitudes towards animals [42,43]. Despite people generally showing high levels of empathy towards domesticated cats and dogs [37,38,39], this measure was not a positive predictor of interspecific CY. In fact, participants with greater empathic concern were *less* likely to yawn during testing, casting doubt on the view that interspecific CY is strongly linked to features of empathy or emotional contagion [16,23,31,32]. Together, these results add to a growing number of psychological studies providing mixed support for a connection between CY and empathy when using self-reported trait measures (reviewed by [17]). 

Based on exploratory analyses of the dataset from Gallup and Wozny [5], a secondary objective of the current study was to further examine the role of gender in interspecific CY among humans. Consistent with most studies in the literature, the current results revealed no overall gender bias in interspecific CY. However, a significant gender × stimulus condition interaction emerged whereby women reported a higher frequency of CY to dog yawns, and men reported a higher frequency of CY to cat yawns. Given that this particular result was not anticipated, we can only speculate as to the mechanisms governing this effect. Prior work has shown, for example, that interactions with pet dogs tend to enhance oxytocin (OT) among female owners, while they lead to no change or decreases in OT among men [62,63]. Moreover, in contrast to human interfaces with dogs, a recent study including only women found no overall increase in OT following interactions with pet cats [64]. Thus, one potential explanation for the female bias in CY to dog stimuli could be differences in OT among participants. However, we are skeptical of this possibility for at least two reasons. First, while OT has been implicated in CY [65,66], studies that have manipulated OT via intranasal administration have failed to yield support for this connection [57,67]. Second, the studies measuring changes in human OT levels were undertaken following real-world interactions with bonded cats and dogs (i.e., pets and their owners), and it seems unlikely that the current procedures of merely viewing static images of unknown animals would elicit similar neurochemical changes. Nevertheless, this represents a potentially fruitful area for future research to explore.

Consistent with similar online studies examining both intra- and interspecific CY in humans [5,7], indices of arousal/fatigue were the strongest predictors of self-reported yawning in the current sample. As expected, sleep duration the night before was negatively correlated with interspecific CY, while tiredness during the study was positively correlated with this response. These findings align with a large body of comparative evidence suggesting that circadian rhythms and internal temperature drive yawning behavior (reviewed by [9]). 

While the current research serves as a replication of interspecific CY in humans and furthers our understanding of the factors contributing to this response, this study has several limitations. First, no measure of pet ownership was captured from the participants. Prior studies suggest that just over half of US households have a pet, with the majority reporting dog and/or cat ownership [68], and thus further work could assess how living and bonding with a pet cat or dog contributes to interspecific CY in these conditions. Another limitation to this study was the relatively small sample of participants, which was reduced primarily due to attentional checks. However, the attentional measures obtained during testing remains a strength of this study, as they ensured (1) high quality data and (2) that visual attention was directed towards the yawning stimuli. That said, the manipulation was not robust, particularly for the binomial outcome. Though common in the literature, the use of one-time measures of yawning to assess the relationship to psychological traits could be considered a further limitation. While we included a self-report measure of affective empathy, we did assess the ability to engage in empathy. Follow-up studies in this area could explore this further while also examining the relationship between interspecific CY and cognitive measures of empathy (i.e., emotion reading and perspective-taking). In addition, further work in this area could attempt to improve the ecological validity of this online experiment, perhaps including live demonstrations of yawning in the laboratory or the use of dynamic (i.e., video) yawn stimuli to enhance the participant response. While self-report CY has proven to be a valid measure of this behavior in psychology [3,56,57], future studies could attempt to capture a combination of both self-report and objective measures of CY.

## 5. Conclusions 

In summary, this study replicates recent findings providing evidence for interspecific CY in humans. By comparing separate conditions for common household pets, we were able to show a slightly greater CY response to yawns of domesticated cats compared to domesticated dogs. Furthermore, when explicitly examining how individual differences in empathic concern and gender influence this response, our findings do not support a strong connection between interspecific CY and empathy or emotional contagion.

## Figures and Tables

**Table 1:** Descriptive Statistics.

Variables					
Stimulus Condition	Gender (Male: Female)	Reported Age (Years)	Prior Sleep (h)	Tiredness (1–10)	Empathic Concern
Control	14:23	35.8 ± 10.4	7.73 ± 2.43	5.70 ± 3.32	23.6 ± 4.91
Cat	23:16	34.5 ± 10.5	7.38 ± 1.04	4.92 ± 3.32	24.7 ± 4.51
Dog	16:11	33.6 ± 9.8	8.59 ± 4.46	5.56 ± 3.26	24.3 ± 5.10
Combined	53:50	34.7 ± 10.2	7.83 ± 2.79	5.37 ± 3.29	24.2 ± 4.79

Data are presented as ratios or M ± SD.

**Table 2:** Binary logistic distribution: estimated parameters (Estimate), standard error (SE), and results of the likelihood ratio tests (*X*^2^).

Variables	Estimate	SE	*df*	*X* ^2^	*p*-Value
Intercept	0.354	0.284	*-*	*-*	*-*
Condition			2	5.67	0.059
Cats ^a^	1.425	0.713			0.046
Dogs ^a^	0.014	0.717			0.985
Gender ^b^	0.106	0.567	1	0.04	0.851
Age	−0.014	0.029	1	0.25	0.616
Sleep	−0.276	0.123	1	8.21	0.004
Tiredness	0.528	0.107	1	37.35	<0.001
Empathic Concern	−0.105	0.055	1	2.40	0.122
Condition × Gender			2	1.24	0.538

Estimate ± SE refers to the difference in the response between the reported level of this categorical predictor and the reference category of the same predictor. Reference categories: ^a^ “Condition (Control)” and ^b^ “Gender (Male)”.

**Table 3:** Poisson distribution: estimated parameters (Estimate), standard error (SE), and results of the likelihood ratio tests (*X*^2^).

Variables	Estimate	SE	*df*	*X* ^2^	*p*-Value
Intercept	0.616	0.085	*-*	*-*	*-*
Condition			2	7.53	0.023
Cats ^a^	0.401	0.154			0.009
Dogs ^a^	0.329	0.170			0.054
Gender ^b^	−0.111	0.130	1	0.74	0.391
Age	0.006	0.005	1	1.21	0.272
Sleep	−0.071	0.026	1	9.58	0.002
Tiredness	0.236	0.024	1	122.53	<0.001
Empathic Concern	−0.032	0.019	1	2.73	0.098
Condition × Gender			2	10.72	0.005

Estimate ± SE refers to the difference in the response between the reported level of this categorical predictor and the reference category of the same predictor. Reference categories: ^a^ “Condition (Control)” and ^b^ “Gender (Male)”.

## Data Availability

The dataset used to generate the results is provided here: https://doi.org/10.7910/DVN/MPU6YF (accessed on 16 March 2023).

## References

[1] Provine R.R. (1986). Yawning as a stereotyped action pattern and releasing stimulus. Ethology.

[2] Provine R.R. (1989). Faces as releasers of contagious yawning: An approach to face detection using normal human subjects. Bull. Psychon. Soc..

[3] Massen J.J., Church A.M., Gallup A.C. (2015). Auditory contagious yawning in humans: An investigation into affiliation and status effects. Front. Psychol..

[4] De Weck M., Perriard B., Annoni J.M., Britz J. (2022). Hearing Someone Laugh and Seeing Someone Yawn: Modality-Specific Contagion of Laughter and Yawning in the Absence of Others. Front. Psychol..

[5] Gallup A.C., Wozny S. (2022). Interspecific Contagious Yawning in Humans. Animals.

[6] Platek S.M., Critton S.R., Myers T.E., Gallup G.G. (2003). Contagious yawning: The role of self-awareness and mental state attribution. Cogn. Brain Res..

[7] Gallup A.C., Kret M.E., Eldakar O.T., Folz J., Massen J.J. (2021). People that score high on psychopathic traits are less likely to yawn contagiously. Sci. Rep..

[8] Anderson J.R., Myowa–Yamakoshi M., Matsuzawa T. (2004). Contagious yawning in chimpanzees. Proc. R. Soc. Lond. Ser. B Biol. Sci..

[9] Gallup A.C. (2022). The causes and consequences of yawning in animal groups. Anim. Behav..

[10] Guggisberg A.G., Mathis J., Schnider A., Hess C.W. (2010). Why do we yawn?. Neurosci. Biobehav. Rev..

[11] Casetta G., Nolfo A.P., Palagi E. (2021). Yawn contagion promotes motor synchrony in wild lions, Panthera leo. Anim. Behav..

[12] Gallup A.C., Meyers K. (2021). Seeing others yawn selectively enhances vigilance: An eye-tracking study of snake detection. Anim. Cogn..

[13] Lehmann H.E. (1979). Yawning: A homeostatic reflex and its psychological significance. Bull. Menn. Clin..

[14] Preston S.D., De Waal F.B. (2002). Empathy: Its ultimate and proximate bases. Behav. Brain Sci..

[15] de Waal F.B., Preston S.D. (2017). Mammalian empathy: Behavioural manifestations and neural basis. Nat. Rev. Neurosci..

[16] Palagi E., Celeghin A., Tamietto M., Winkielman P., Norscia I. (2020). The neuroethology of spontaneous mimicry and emotional contagion in human and non-human animals. Neurosci. Biobehav. Rev..

[17] Massen J.J., Gallup A.C. (2017). Why contagious yawning does not (yet) equate to empathy. Neurosci. Biobehav. Rev..

[18] Zentall T.R. (2006). Imitation: Definitions, evidence, and mechanisms. Anim. Cogn..

[19] Yoon J.M., Tennie C. (2010). Contagious yawning: A reflection of empathy, mimicry, or contagion?. Anim. Behav..

[20] Gallup A.C. (2021). On the link between emotional contagion and contagious yawning. Neurosci. Biobehav. Rev..

[21] Palagi E., Celeghin A., Tamietto M., Winkielman P., Norscia I. (2022). Disentangling attentional and affective contribution to contagious yawning. Neurosci. Biobehav. Rev..

[22] Joly-Mascheroni R.M., Senju A., Shepherd A.J. (2008). Dogs catch human yawns. Biol. Lett..

[23] Silva K., Bessa J., De Sousa L. (2012). Auditory contagious yawning in domestic dogs (*Canis familiaris*): First evidence for social modulation. Anim. Cogn..

[24] Xu X., Zuo X., Wang X., Han S. (2009). Do you feel my pain? Racial group membership modulates empathic neural responses. J. Neurosci..

[25] Mathur V.A., Harada T., Lipke T., Chiao J.Y. (2010). Neural basis of extraordinary empathy and altruistic motivation. NeuroImage.

[26] Avenanti A., Sirigu A., Aglioti S.M. (2010). Racial bias reduces empathic sensorimotor resonance with other-race pain. Curr. Biol..

[27] Romero T., Konno A., Hasegawa T. (2013). Familiarity bias and physiological responses in contagious yawning by dogs support link to empathy. PLoS ONE.

[28] Madsen E.A., Persson T. (2013). Contagious yawning in domestic dog puppies (*Canis lupus familiaris*): The effect of ontogeny and emotional closeness on low-level imitation in dogs. Anim. Cogn..

[29] Neilands P., Claessens S., Ren I., Hassall R., Bastos A.P., Taylor A.H. (2020). Contagious yawning is not a signal of empathy: No evidence of familiarity, gender or prosociality biases in dogs. Proc. R. Soc. B.

[30] Campbell M.W., de Waal F.B. (2011). Ingroup-outgroup bias in contagious yawning by chimpanzees supports link to empathy. PLoS ONE.

[31] Campbell M.W., de Waal F.B. (2014). Chimpanzees empathize with group mates and humans, but not with baboons or unfamiliar chimpanzees. Proc. R. Soc. B Biol. Sci..

[32] Pedruzzi L., Aychet J., Le Vern L., Maglieri V., Rossard A., Lemasson A., Palagi E. (2022). Familiarity modulates both intra-and interspecific yawn contagion in red-capped mangabeys. Sci. Rep..

[33] Rossman Z.T., Padfield C., Young D., Hart B.L., Hart L.A. (2020). Contagious yawning in African elephants (Loxodonta fricana): Responses to other elephants and familiar humans. Front. Vet. Sci..

[34] Harrison M.A., Hall A.E. (2010). Anthropomorphism, empathy, and perceived communicative ability vary with phylogenetic relatedness to humans. J. Soc. Evol. Cult. Psychol..

[35] Westbury H.R., Neumann D.L. (2008). Empathy-related responses to moving film stimuli depicting human and non-human animal targets in negative circumstances. Biol. Psychol..

[36] Miralles A., Raymond M., Lecointre G. (2019). Empathy and compassion toward other species decrease with evolutionary divergence time. Sci. Rep..

[37] Kurdek L.A. (2008). Pet dogs as attachment figures. J. Soc. Pers. Relatsh..

[38] Kurdek L.A. (2009). Pet dogs as attachment figures for adult owners. J. Fam. Psychol..

[39] Vitale K.R., Behnke A.C., Udell M.A. (2019). Attachment bonds between domestic cats and humans. Curr. Biol..

[40] Gallup A.C., Massen J.J. (2016). There is no difference in contagious yawning between men and women. R. Soc. Open Sci..

[41] Norscia I., Demuru E., Palagi E. (2016). She more than he: Gender bias supports the empathic nature of yawn contagion in Homo sapiens. R. Soc. Open Sci..

[42] Taylor N., Signal T.D. (2005). Empathy and attitudes to animals. Anthrozoös.

[43] Signal T.D., Taylor N. (2007). Attitude to animals and empathy: Comparing animal protection and general community samples. Anthrozoös.

[44] Franzen A., Mader S., Winter F. (2018). Contagious yawning, empathy, and their relation to prosocial behavior. J. Exp. Psychol. Gen..

[45] Gottfried J., Lacinová L., Širůček J. (2015). Contagious yawning and empathy. Exp. Psycholol..

[46] Haker H., Rössler W. (2009). Empathy in schizophrenia: Impaired resonance. Eur. Arch. Psychiatry Clin. Neurosci..

[47] Bartholomew A.J., Cirulli E.T. (2014). Individual variation in contagious yawning susceptibility is highly stable and largely unexplained by empathy or other known factors. PLoS ONE.

[48] Perri A.R., Feuerborn T.R., Frantz L.A., Larson G., Malhi R.S., Meltzer D.J., Witt K.E. (2021). Dog domestication and the dual dispersal of people and dogs into the Americas. Proc. Natl. Acad. Sci. USA.

[49] Kujala M.V., Somppi S., Jokela M., Vainio O., Parkkonen L. (2017). Human empathy, personality and experience affect the emotion ratings of dog and human facial expressions. PLoS ONE.

[50] Abbey J.D., Meloy M.G. (2017). Attention by design: Using attention checks to detect inattentive respondents and improve data quality. J. Oper. Manag..

[51] Goodman J.K., Cryder C.E., Cheema A. (2013). Data collection in a flat world: The strengths and weaknesses of Mechanical Turk samples. J. Behav. Decis. Mak..

[52] Yarrish C., Groshon L., Mitchell J., Appelbaum A., Klock S., Winternitz T., Friedman-Wheeler D.G. (2019). Finding the signal in the noise: Minimizing responses from bots and inattentive humans in online research. Behav. Ther..

[53] Helt M.S., Sorensen T.M., Scheub R.J., Nakhle M.B., Luddy A.C. (2021). Patterns of contagious yawning and itching differ amongst adults with autistic traits vs. psychopathic traits. Front. Psychol..

[54] Davis M.H. (1980). A multidimensional approach to individual differences in empathy. JSAS Cat. Sel. Doc. Psychol..

[55] Herzog H., Grayson S., McCord D. (2015). Brief measures of the animal attitude scale. Anthrozoös.

[56] Greco M., Baenninger R. (1989). Self-report as a valid measure of yawning in the laboratory. Bull. Psychon. Soc..

[57] Gallup A.C., Church A.M. (2015). The effects of intranasal oxytocin on contagious yawning. Neurosci. Lett..

[58] Zilli I., Giganti F., Salzarulo P. (2007). Yawning in morning and evening types. Physiol. Behav..

[59] Giganti F., Zilli I. (2011). The daily time course of contagious and spontaneous yawning among humans. J. Ethol..

[60] Faul F., Erdfelder E., Lang A.G., Buchner A. (2007). G* Power 3: A flexible statistical power analysis program for the social, behavioral, and biomedical sciences. Behav. Res. Methods.

[61] (2021). The Jamovi Project Jamovi (Version 1.6) [Computer Software]. https://www.jamovi.org.

[62] Nagasawa M., Mitsui S., En S., Ohtani N., Ohta M., Sakuma Y., Onaka T., Mogi K., Kikusui T. (2015). Oxytocin-gaze positive loop and the coevolution of human-dog bonds. Science.

[63] Miller S.C., Kennedy C.C., DeVoe D.C., Hickey M., Nelson T., Kogan L. (2009). An examination of changes in oxytocin levels in men and women before and after interaction with a bonded dog. Anthrozoös.

[64] Johnson E.A., Portillo A., Bennett N.E., Gray P.B. (2021). Exploring women’s oxytocin responses to interactions with their pet cats. PeerJ.

[65] Mariscal M.G., Oztan O., Rose S.M., Libove R.A., Jackson L.P., Sumiyoshi R.D., Trujillo T., Carson D., Phillips J., Garner J. (2019). Blood oxytocin concentration positively predicts contagious yawning behavior in children with autism spectrum disorder. Autism Res..

[66] Norscia I., Agostini L., Moroni A., Caselli M., Micheletti-Cremasco M., Vardé C., Palagi E. (2021). Yawning Is More Contagious in Pregnant Than Nulliparous Women: Naturalistic and Experimental Evidence. Hum. Nat..

[67] Kis A., Tóth K., Kanizsár O., Topál J. (2020). The effect of oxytocin on yawning by dogs (*Canis familiaris*) exposed to human yawns. Appl. Anim. Behav. Sci..

[68] Hawes S.M., Hupe T.M., Gandenberger J., Saucedo M., Arrington A., Morris K.N. (2022). Detailed assessment of pet ownership rates in four underserved urban and rural communities in the United States. J. Appl. Anim. Welf. Sci..

